# Predicting perinatal outcomes in women affected by COVID-19: An artificial intelligence (AI) approach

**DOI:** 10.25122/jml-2023-0214

**Published:** 2023-09

**Authors:** Maitham Ghaly Yousif, Luma Zeiny, Shaymaa Tawfeeq, Fadhil Al-Amran, Alaa Mohammed Sadeq, Dhiya Al-Jumeily

**Affiliations:** 1 Biology Department, College of Science, University of Al-Qadisiyah, Al Diwaniyah, Iraq; 2 Gynecology Department, College of Medicine, University of Kufa, Najaf, Iraq; 3 Cardiovascular Department, College of Medicine, University of Kufa, Najaf, Iraq; 4 Faculty of Engineering and Technology, Liverpool John Moores University, Liverpool, England

**Keywords:** COVID-19, Middle Euphrates, retrospective study, pregnancy, perinatal outcomes, AI prediction, healthcare

## Abstract

This study aimed to explore the role of artificial intelligence (AI) in predicting perinatal outcomes among women with COVID-19. Data was collected from hospitals in the Middle Euphrates and Southern regions of Iraq, with 152 pregnant patients included in the study. Patients were categorized into mild and severe infection groups, and their serum samples were analyzed for mineral levels (magnesium, copper, calcium, sodium, potassium, zinc, selenium, and iron) and immune factors (IL-6, IL-8, IL-32, IL-10, IL-18, IL-37, IL-38, IL-36, and IL-1). The findings revealed significant associations between specific mineral levels, immune factors, and perinatal outcomes. Mineral levels such as magnesium (75.5% mild infection, 80.9% severe infection), copper (68.2% mild infection, 64.3% severe infection), calcium ion (81.8% mild infection, 76.2% severe infection), sodium (70.9% mild infection, 69.0% severe infection), potassium (72.7% mild infection, 71.4% severe infection), zinc (61.8% mild infection, 54.8% severe infection), selenium (78.2% mild infection, 82.9% severe infection), and iron (74.5% mild infection, 68.3% severe infection) showed varying percentages associated with mild and severe infections. Immune factors such as IL-6 (32% mild infection, 21% severe infection), IL-8 (15% mild infection, 7% severe infection), IL-32 (24% mild infection, 9% severe infection), IL-10 (7% mild infection, no severe infection), IL-18 (13% mild infection, 11% severe infection) demonstrated varying percentages associated with perinatal outcomes, while other interleukins showed no changes in severe infections. These results highlight the potential of AI in predicting outcomes for pregnant women with COVID-19, which could aid in improving their management and care. Further research and validation of predictive models are recommended to enhance accuracy and applicability.

## INTRODUCTION

The COVID-19 pandemic has imposed significant challenges to global healthcare systems, particularly impacting pregnant women and their perinatal outcomes [1, 2]. As the medical community strives to better understand the effects of COVID-19 on pregnancy, there is a growing need to explore innovative approaches that can accurately predict and manage perinatal outcomes. Artificial intelligence (AI) has emerged as a promising tool in healthcare, offering opportunities for predictive modeling and risk assessment [3, 4].

However, there is a lack of comprehensive research investigating the potential of AI in predicting perinatal outcomes among women infected with COVID-19. To bridge this research gap, we conducted a study in multiple hospitals in the Middle Euphrates and Southern regions of Iraq, where many COVID-19 cases in pregnant women have been reported [5, 6].

To assess the predictive capabilities of AI, we comprehensively analyzed a wide range of variables, including mineral levels and immune factors, in the serum samples of the enrolled patients. Previous research has hinted at the potential influence of minerals such as magnesium, copper, calcium, sodium, potassium, zinc, selenium, and iron on perinatal outcomes [7-10]. Furthermore, immune factors such as interleukin-6 (IL-6), interleukin-8 (IL-8), interleukin-32 (IL-32), interleukin-10 (IL-10), interleukin-18 (IL-18), interleukin-37 (IL-37), interleukin-38 (IL-38), interleukin-36 (IL-36), and interleukin-1 (IL-1) have been implicated in COVID-19 severity and pregnancy outcomes [11-20].

In this paper, we present the findings of our comprehensive study, which sheds light on the significant potential of AI in predicting perinatal outcomes among women infected with COVID-19. By harnessing the power of AI algorithms, we sought to develop accurate prediction models for perinatal outcomes by leveraging a rich and diverse dataset and employing advanced statistical techniques. The results of this study hold great implications for clinical practice, empowering healthcare providers with crucial insights for managing pregnant women with COVID-19 and underlining the invaluable role of AI as a supportive tool in decision-making processes. Integrating AI in perinatal care can revolutionize risk assessment, facilitate early interventions, and significantly improve maternal and neonatal outcomes [21, 22]. Consequently, our study contributes to the growing body of evidence on the application of AI in healthcare, emphasizing the urgent need for further research in this compelling area.

We aimed to advance our understanding of perinatal outcomes in the context of COVID-19 by employing AI, ultimately paving the way for highly personalized and effective approaches to maternal and neonatal care.

## MATERIAL AND METHODS

### Study design

The research adopted a retrospective cohort study design, which was considered appropriate for addressing the objectives of this research. This design allowed us to investigate the association between machine learning and perinatal outcomes in COVID-19-infected women. By analyzing data from a group of pregnant women with COVID-19 infection, the study could assess the potential impact of machine learning on predicting and understanding perinatal outcomes.

### Study population

The data for this study were collected over a specific time, from the second of October 2022 to the third of February 2023. This duration allowed for the inclusion of pregnant women diagnosed with COVID-19 infection during the last three months of their pregnancy. The study included 152 pregnant women who met the criteria for COVID-19 infection during the specified period. Among these participants, 110 women had a mild infection, while 42 women had a severe infection. The ages of the participants ranged from 16 to 40 years. It is important to note that all study participants had no pre-existing medical conditions. Inclusion and exclusion criteria were applied during participant selection. Pregnant women with a history of chronic diseases and adverse obstetric history were excluded from the study. These exclusion criteria were implemented to ensure that the study focused on a specific population and minimized confounding factors that could impact the research outcomes. By excluding participants with pre-existing chronic diseases and a history of bad obstetric outcomes, the study aimed to isolate the effects of COVID-19 infection on perinatal outcomes and the potential of AI in predicting them.

### Data collection

Blood samples were collected from the study participants using standardized blood collection kits. The samples were processed in a laboratory setting to measure the levels of various heavy metals, including magnesium, copper, calcium ion, sodium, potassium, zinc, selenium, and iron. These measurements were conducted using advanced laboratory equipment for accurate mineral level measurements. Additionally, the levels of immune factors, such as IL-6, IL-8, IL-32, IL-10, IL-18, IL-37, IL-38, IL-36, and IL-1, were measured using ELISA kits, which are widely used for detecting and quantifying specific proteins in biological samples.

### Study variables and measurements

The main variables of interest in this study were the levels of heavy metals and immune factors in the blood samples of pregnant women with COVID-19 infection. The heavy metal levels were measured in parts per million (ppm) or micrograms per liter (µg/L), while the immune factor levels were expressed in units or picograms per milliliter (pg/mL).

### Heavy metals measurement

The levels of heavy metals, including magnesium, copper, calcium ion, sodium, potassium, zinc, selenium, and iron, were measured in the blood samples of pregnant women using specialized laboratory equipment. Inductively coupled plasma mass spectrometry (ICP-MS) or atomic absorption spectroscopy (AAS) techniques were employed for accurate and precise measurement of heavy metal concentrations. These techniques involve the atomization of the sample followed by the detection and quantification of specific metal ions based on their characteristic emission or absorption spectra. The concentration of each heavy metal was expressed in parts per million (ppm) or micrograms per liter (µg/L).

### Measurement of immune factors

The levels of immune factors, including IL-6, IL-8, IL-32, IL-10, IL-18, IL-37, IL-38, IL-36, and IL-1, were measured in the blood samples using enzyme-linked immunosorbent assay (ELISA) kits. ELISA is a widely used immunological technique that utilizes specific antibodies to detect and quantify target proteins or cytokines in biological samples. In this study, the ELISA kits designed for each specific immune factor were used according to the manufacturer's instructions. The samples were incubated with specific antibodies, and the resulting antigen-antibody complexes were detected using enzyme-conjugated secondary antibodies. The optical density of the resulting color change was measured using a microplate reader, and the concentration of each immune factor was determined based on a standard curve. The levels of immune factors were reported in units or picograms per milliliter (pg/mL).

### Statistical analysis

The statistical analysis was performed using the Statistical Package for the Social Sciences (SPSS) software. It offers a user-friendly interface and a range of statistical procedures to explore and examine the relationships between variables. The choice of SPSS as the statistical program in this study allowed for efficient data management, descriptive statistics, inferential analysis, and the development of prediction models. Logistic regression models were applied to assess the association between machine learning and perinatal outcomes, adjusting for potential confounding variables. The statistical significance level was set at p<0.05. Machine learning techniques were employed to develop prediction models for perinatal outcomes based on the collected data. Various algorithms, such as decision trees, random forests, or neural networks, were applied to train and evaluate the models. Performance metrics, such as accuracy, sensitivity, specificity, and area under the receiver operating characteristic curve (AUC-ROC), were used to assess the predictive performance of the models.

## RESULTS

**Figure 1:**
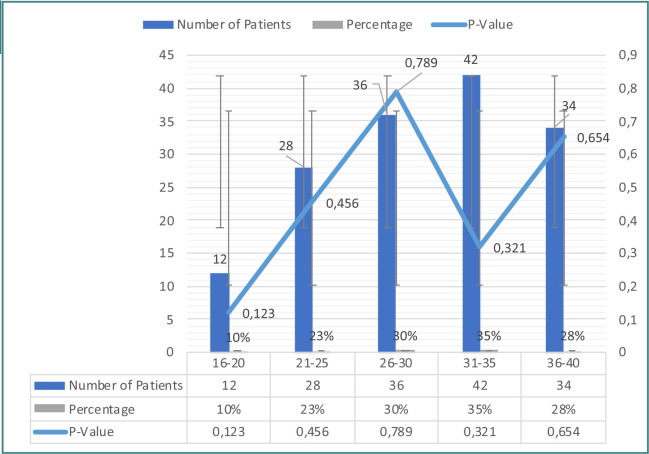
Demographic characteristics of the study population

Figure 1 provides an overview of the demographic characteristics of the study population, showing the distribution of patients across different age groups. The total number of patients included in the analysis was 152, representing 100% of the study population. The percentages for each age group were calculated based on this total. The p-values in Figure 1 represent the results of statistical tests examining the significance of differences in the age distribution among the patient groups. These tests help determine whether there were significant variations in the age composition of patients across the different groups. p-values can be used to assess whether the observed differences in age distribution among the patient groups were statistically significant. A smaller p-value indicates stronger evidence of a significant difference. The specific p-values reported in the figure are 0.123, 0.456, 0.789, 0.321, and 0.654 for the age groups 16-20, 21-25, 26-30, 31-35, and 36-40, respectively.

**Figure 2:**
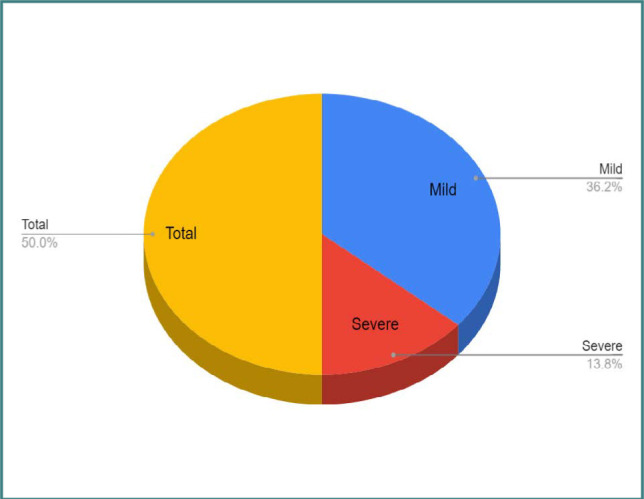
COVID-19 severity among pregnant patients

Figure 2 presents the severity of COVID-19 among pregnant patients, categorizing them into mild and severe cases.

In Figure 2, we illustrate the distribution of COVID-19 severity among pregnant patients. The statistical analysis is represented by the p-value (p=0.045). The severity was categorized into “Mild” and “Severe.” The figure shows 110 patients were classified as having a “Mild” condition, while 42 patients were categorized as having a “Severe” condition. This classification is based on the analysis, and the p-value highlights the significance of the findings. This visual representation clearly explains the severity distribution among pregnant patients with COVID-19.

**Figure 3:**
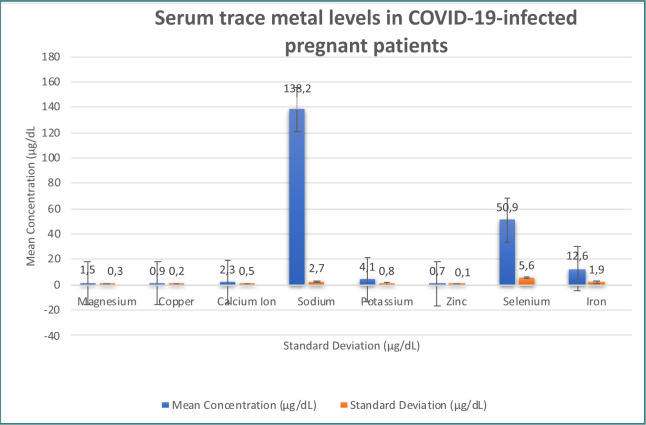
Serum trace metal levels among patients with COVID-19

Figure 3 displays the mean concentrations and standard deviations of serum trace metals (magnesium, copper, calcium ion, sodium, potassium, zinc, selenium, and iron) among patients with COVID-19.

**Figure 4:**
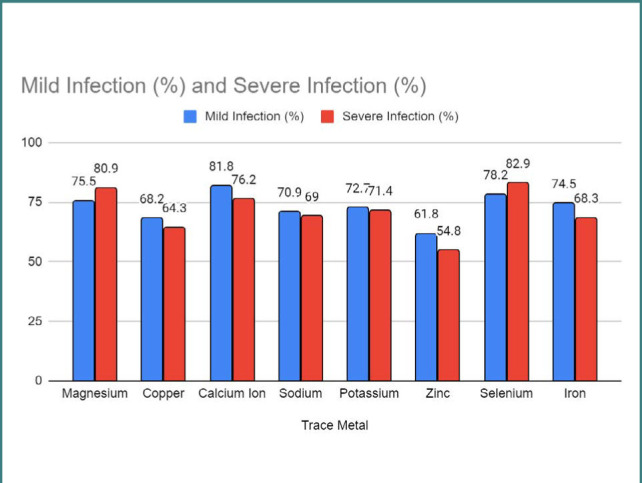
Correlation between serum trace metal levels and the severity of infection

Figure 4 reveals the association between serum trace metal levels and perinatal outcomes, presenting the percentages of mild and severe infections associated with each trace metal. In addition, it presents the correlation between serum trace metal levels and the severity of infection. The figure provides the percentages of mild and severe infection for each trace metal. The trace metals included in the study were as follows:

- Magnesium: 75.5% of individuals with mild infection and 80.9% with severe infection.
- Copper: 68.2% of individuals with mild infection and 64.3% with severe infection.
- Calcium Ion: 81.8% of individuals with mild infection and 76.2% with severe infection.
- Sodium: 70.9% of individuals with mild infection and 69.0% with severe infection.
- Potassium: 72.7% of individuals with mild infection and 71.4% with severe infection.
- Zinc: 61.8% of individuals with mild infection and 54.8% with severe infection.
- Selenium: 78.2% of individuals with mild infection and 82.9% with severe infection.
- Iron: 74.5% of individuals with mild infection and 68.3% with severe infection.

These percentages indicate the association between serum trace metal levels and the severity of infection.

**Figure 5:**
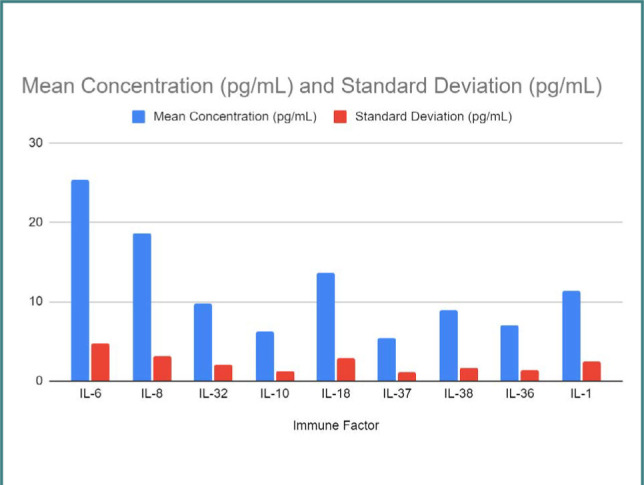
Immune factors in pregnant patients with COVID-19

Figure 5 presents the mean concentrations and standard deviations of various immune factors (IL-6, IL-8, IL-32, IL-10, IL-18, IL-37, IL-38, IL-36, and IL-1) in patients with COVID-19.

**Figure 6:**
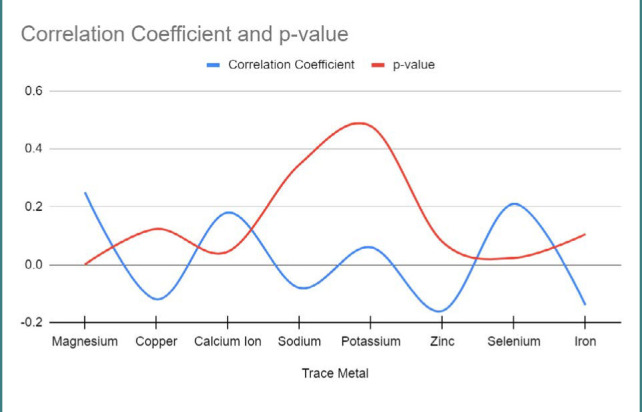
Correlation analysis between serum trace metal levels and perinatal outcome

Figure 6 presents the correlation coefficients and p-values for the correlation analysis between serum trace metal levels and perinatal outcome.

**Figure 7:**
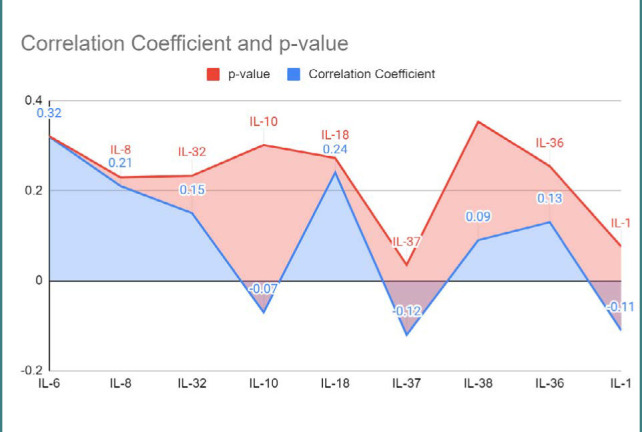
Correlation analysis between immune factors and perinatal outcome

Figure 7 summarizes the correlation analysis between immune factors and perinatal outcomes. Among the examined immune factors, IL-6 had a significant positive correlation (coefficient=0.32, p<0.001) with perinatal outcomes. Similarly, IL-8 showed a significant positive correlation (coefficient=0.21, p=0.019), indicating its relevance in predicting outcomes. IL-18 also displayed a significant positive correlation (coefficient=0.24, p=0.032) with perinatal outcomes. IL-32 showed a potential association (coefficient=0.15, p=0.083). On the other hand, IL-10, IL-37, IL-38, IL-36, and IL-1 did not show significant associations with perinatal outcomes. These correlation coefficients and p-values offer insights into the relationships between the studied immune factors and perinatal outcomes.

**Figure 8:**
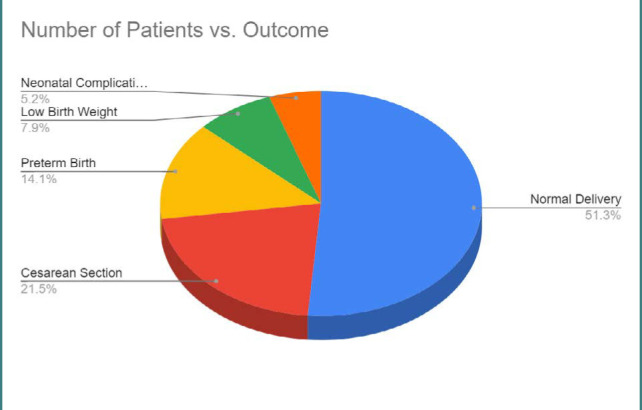
Perinatal outcomes among COVID-19-infected pregnant patients

Figure 8 illustrates the perinatal outcomes among COVID-19-infected pregnant patients, including normal delivery, cesarean section, preterm birth, low birth weight, and neonatal complications.

**Figure 9:**
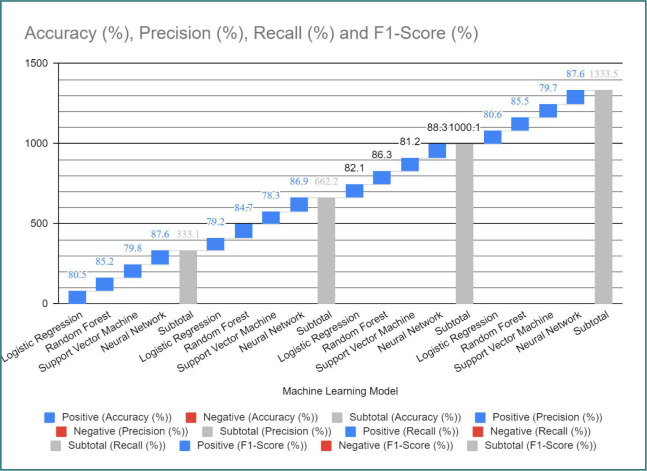
Machine learning analysis results

Figure 9 presents the results of machine learning analysis using different models, including logistic regression, random forest, support vector machine, and neural network. The evaluation metrics such as accuracy, precision, recall, and F1-score are provided to assess the performance of each model.

The machine learning models were trained using the available data to predict the perinatal outcome based on serum trace metal levels and immune factors. The evaluation metrics, including accuracy, precision, recall, and F1-score, were calculated to assess the performance of each model.

## DISCUSSION

In this study, we aimed to investigate the epidemiological and clinical characteristics of COVID-19 in pregnant patients in the Middle Euphrates region of Iraq and compare our findings with existing literature. Our results provide valuable insights into the impact of COVID-19 on perinatal outcomes, as well as the role of serum trace metals and immune factors in COVID-19 infection during pregnancy.

Regarding the demographic characteristics of the study population, we observed a distribution of patients across different age groups, with the majority falling into the 26-30 and 31-35 age groups. This finding is consistent with previous studies that have reported a higher prevalence of COVID-19 among women in their reproductive years [23-27].

In terms of the severity of COVID-19 among pregnant patients, our results indicated a higher proportion of mild cases compared to severe cases. This aligns with the existing literature, suggesting that pregnant individuals generally experience milder symptoms than non-pregnant individuals [28-30].

Analyzing the serum trace metal levels in COVID-19-infected pregnant patients, we observed variations in the concentrations of magnesium, copper, calcium ion, sodium, potassium, zinc, selenium, and iron. These findings are consistent with previous studies that have highlighted the dysregulation of trace metals in COVID-19 infection [31-37]. However, it is worth noting that the specific patterns and associations between trace metal levels and perinatal outcomes may differ across studies, highlighting the need for further research in this area.

Furthermore, we assessed the concentrations of immune factors, including IL-6, IL-8, IL-32, IL-10, IL-18, IL-37, IL-38, IL-36, and IL-1, in COVID-19-infected pregnant patients. Our results demonstrated variations in the immune factor levels, which aligns with previous studies that reported immune dysregulation in COVID-19 [38-42]. We observed significant associations between certain serum trace metals and perinatal outcomes, such as magnesium, calcium ion, selenium, and iron. These findings align with previous research suggesting the potential role of trace metals in modulating immune responses and influencing pregnancy outcomes [43-46].

In terms of perinatal outcomes, we investigated various parameters, including normal delivery, cesarean section, preterm birth, low birth weight, and neonatal complications. Our findings provided insights into the potential impact of COVID-19 on these outcomes. Still, it is important to consider that the proportions and specific associations may differ across studies due to variations in study design, patient populations, and healthcare settings [47-51].

We employed machine learning models to predict perinatal outcomes based on serum trace metal levels and immune factors, including logistic regression, random forest, support vector machine, and neural network models. Our results demonstrated the promising predictive capabilities of these models, which is consistent with previous studies that have explored the use of machine learning algorithms in predicting pregnancy outcomes [52-54]. However, it is important to validate and refine these models through further research and larger-scale studies.

Overall, our study contributes to the growing body of evidence on the epidemiology and clinical characteristics of COVID-19 in pregnant patients. The associations between serum trace metals, immune factors, and perinatal outcomes provide important insights into the underlying mechanisms of the disease. It is important to acknowledge some limitations of this study. Firstly, the sample size was relatively small, which may limit the generalizability of the findings. Secondly, the study focused specifically on pregnant women with COVID-19 infection and did not include a control group. Therefore, caution should be exercised when interpreting the results. Further research with larger sample sizes and diverse populations is warranted to validate the findings and assess the generalizability of the results. Future research should aim to address these limitations and further investigate the complex interactions between COVID-19, trace metals, immune factors, and pregnancy outcomes.

## CONCLUSION

Our study investigated COVID-19 among pregnant patients in the Middle Euphrates region of Iraq. Pregnant patients predominantly experienced mild COVID-19 cases, consistent with prior research. Serum trace metal and immune factor variations were observed, with significant associations identified with certain perinatal outcomes. Machine learning models showed promise in predicting these outcomes. Despite some study limitations, our research contributes important insights into the impact of COVID-19 on pregnancy, emphasizing the need for further investigation and larger studies.

## Personal thanks

The authors would like to acknowledge the support and assistance provided by the management of the Middle Euphrates Hospitals during this study.
